# Response of Human Glioblastoma Cells to Vitamin B12 Deficiency: A Study Using the Non-Toxic Cobalamin Antagonist

**DOI:** 10.3390/biology10010069

**Published:** 2021-01-19

**Authors:** Zuzanna Rzepka, Jakub Rok, Mateusz Maszczyk, Artur Beberok, Justyna Magdalena Hermanowicz, Dariusz Pawlak, Dorota Gryko, Dorota Wrześniok

**Affiliations:** 1 Department of Pharmaceutical Chemistry, Faculty of Pharmaceutical Sciences in Sosnowiec, Medical University of Silesia in Katowice, Jagiellońska 4, 41-200 Sosnowiec, Poland; zrzepka@sum.edu.pl (Z.R.); jrok@sum.edu.pl (J.R.); d200888@365.sum.edu.pl (M.M.); abeberok@sum.edu.pl (A.B.); 2 Department of Pharmacodynamics, Medical University of Bialystok, Mickiewicza 2C, 15-222 Bialystok, Poland; justyna.hermanowicz@umb.edu.pl (J.M.H.); dariuszpawlak@poczta.onet.pl (D.P.); 3 Institute of Organic Chemistry, Polish Academy of Science, Kasprzaka 44/52, 01-224 Warsaw, Poland; dorota.gryko@icho.edu.pl

**Keywords:** cobalamin, vitamin B12, glioblastoma, vitamin B12 deficiency, antivitamins, cobalamin antagonists

## Abstract

**Simple Summary:**

The most important biological function of vitamin B12 (cobalamin) is to accomplish DNA synthesis, which is necessary for cell division. Cobalamin deficiency may be especially acute for rapidly dividing cells, such as glioblastoma cells. Therefore, cobalamin antagonists offer a medicinal potential for developing anti-glioma agents. In the present study, we revealed, for the first time, that the induction of cobalamin deficiency by vitamin B12 antagonist with affinity to key transporter of cobalamins, inhibited glioblastoma cells growth and promoted cell cycle arrest. The effect was observed for non-toxic concentration of the agent, as demonstrated on zebrafish. Moreover, as compared to our previous study, the cytostatic effect of the agent was more pronounced in glioblastoma cells than in normal astrocytes. We believe that the study may become the basis for further in vitro and in vivo experiments concerning cobalamin deprivation as a potential therapeutic strategy for glioblastoma.

**Abstract:**

The most important biological function of vitamin B12 is to accomplish DNA synthesis, which is necessary for cell division. Cobalamin deficiency may be especially acute for rapidly dividing cells, such as glioblastoma cells. Therefore, cobalamin antagonists offer a medicinal potential for developing anti-glioma agents. In the present study, we developed an in vitro model of cobalamin deficiency in glioblastoma cells. Long-term treatment of cells with the cobalamin analogue, hydroxycobalamin [*c*-lactam] (HCCL) was applied to induce an increase of hypocobalaminemia biomarker. Cytometric assays demonstrated that vitamin B12 promoted glioblastoma cells proliferation, whereas the treatment of cells with HCCL caused a dramatic inhibition of cell proliferation and an induction of cell cycle arrest at the G2/M phase. Vitamin B12 counteracted all the observed effects of HCCL. In the in silico study, we characterized the molecular interactions between HCCL and transcobalamin II (TCII). We have demonstrated that HCCL shares similar interactions with TCII as naturally occurring cobalamins and therefore may act as a competitive inhibitor of this key transporter protein. We assessed the impact of HCCL on the mortality or developmental malformations of zebrafish embryos. Collectively, our findings suggest that the use of cobalamin transport antagonists as potential anti-glioma agents would be worth exploring further.

## 1. Introduction

Glioblastoma (GB) is one of the deadliest neoplasms, showing a five-year survival rate of 4–5%. The mean survival of patients with GB remains below 20 months despite numerous advances in medical research [1,2,3]. Several factors may limit the effectiveness of current treatments for GB, including molecular heterogeneity, tumor cell invasion, and the multidrug resistance proteins of the blood-brain barrier, which prevents the accumulation of xenobiotics within the central nervous system [4]. Thus, new therapeutic strategies are needed to circumvent these limitations.

During the development of new anticancer agents, particular attention should be paid to the differences between normal and cancer cells, as potential targets. Then the effectiveness and safety of the treatment might be expected. One of the characteristics of cancer cells is the high demand for cobalamin (Cbl) also known as vitamin B12 (B12) [5,6]. Two proteins involved in the cellular uptake of cobalamin: transcobalamin II (TCII) and transcobalamin II receptor (TCblR/CD320) have been explored as potential tumor biomarkers as their expression is elevated in cancer cells [5,7].

Cbl is an essential vitamin for the human organism, stored in the liver. Because body stores of Cbl exceed several milligrams, insufficient intake, or malabsorption of vitamin B12 for more than five years usually is required to produce clinical symptoms of its deficiency, including neurological disorders. Cbl occurs naturally as methylcobalamin, hydroxocobalamin, and adenosylcobalamin [8]. Receptor-mediated endocytosis of B12 involves TCII (a plasma protein that binds Cbl) and TCblR-cell surface receptor that specifically binds TCII saturated with Cbl [9]. Once inside a cell, B12 acts as a cofactor for methionine synthase (MS) and methylmalonyl-coenzyme A mutase. In the MS reaction a methyl group is transferred from methyltetrahydrofolate to homocysteine (HCY), with methylcobalamin as a coenzyme. The resulting tetrahydrofolate can then be recycled to the folic acid pool and made available for the production of methylenetetrahydrofolate, a form required for de novo synthesis of thymidylate, which is essential for DNA replication and repair. In vitamin B12 deficiency, folate is “trapped” in the methyl-form, and thus cell proliferation is disturbed, and DNA impairment may occur. Moreover, the common cellular consequence of cobalamin deficit is an accumulation of HCY and reduced synthesis of methionine and S-adenosylmethionine (SAM). HCY accumulation can induce oxidative stress, apoptosis and homocysteinylation of functional proteins whereas SAM is the methyl donor that is required for epigenetic reactions, including DNA methylation [10].

It is well established that rapidly proliferating cells such as cancer cells are much stronger consumers of B12 or folate (vitamin B9) than healthy cells and are particularly prone to deficiency of these vitamins [6,10,11]. For this reason, antivitamins B9 or antivitamins B12 may induce antiproliferative effects in neoplastic cells having great potential for applications in oncology. Methotrexate and pemetrexed, two drugs that originated from a slight structural modification of vitamin B9, are currently used to treat human cancers. Contrariwise, no antivitamin B12 derivative has reached the market yet. It was noted that MS shows low specificity for structurally modified B12 derivatives, thus suggesting that the most attractive and reasonable targets are the proteins involved in the uptake, delivery, and metabolism of B12 [6]. In vitro and animal studies have shown the non-functional structural analogue of vitamin B12, hydroxycobalamin [*c*-lactam] (HCCL), to be an effective antivitamin B12 [12,13,14,15,16,17,18,19]. A target protein for HCCL has not yet been identified. It was suggested that the agent may block the intracellular uptake and/or subsequent metabolic conversion of cobalamins [6].

Previous in vitro studies have indicated that vitamin B12 deficiency significantly affects neuroblastoma cells, reduces proliferation, promotes differentiation [20] and induces endoplasmic reticulum stress [21]. However, so far, no studies have been conducted on the effect of HCCL or other Cbl antagonists on brain cancer cells growth and survival.

Despite extensive knowledge of the biological functions of B12, the impact of Cbl status on glioblastoma growth is still enigmatic. We hypothesize that Cbl deficit may restrict excessive cell proliferation and lead to DNA damage [22]. To address this hypothesis we developed a stable in vitro model of hypocobalaminemia in glioblastoma cells by the long-term treatment with HCCL. The dysfunctions in cellular metabolism due to vitamin B12 deficiency result in the accumulation of HCY [23] and its export to the culture medium [18,24]. Thus, to determine the time after which cells cultured with HCCL were Cbl-deficient, we monitored the extracellular HCY levels. Then we investigated the effects of B12 status on cell viability, proliferation, and cell cycle progression. Since the zebrafish (*Danio rerio*) is an advantageous in vivo model system for preclinical drug discovery and screening, we examined the effect of HCCL on mortality and development of zebrafish embryos. Finally, to shed light on the mechanism of action of HCCL, we performed in silico analysis to investigate the capacity of binding the HCCL to TCII and compared it with the parameters determined for naturally occurring forms of B12.

## 2. Materials and Methods

### 2.1. Materials

Amphotericin B; Bovine Serum Albumin (BSA); Penicillin G were purchased from Sigma-Aldrich Inc. (St. Luis, MO, USA). NC-Slides A8 and A2, Solution 3 (1 μg/mL DAPI, 0.1% triton X-100 in PBS) and Via1-Cassettes were obtained from ChemoMetec (Lillerød, Denmark). Dulbecco’s phosphate-buffered saline; Trypsin/EDTA solution were purchased from Thermo Fisher Scientific (Waltham, MA, USA). Growth medium DMEM and fetal bovine serum were acquired from CytoGen (Zgierz, Poland). Neomycin sulfate was obtained from Amara (Kraków, Poland). Vitamin B12 was purchased from Polfa Warszawa S.A. (Warszawa, Poland). Human Hcy ELISA Kit was purchased from Abbexa (Cambridge, UK). The remaining chemicals were purchased from POCH S.A. (Gliwice, Poland). Hydroxycobalamin [*c*-lactam] was synthesized by Prof. Dorota Gryko (Institute of Organic Chemistry, Polish Academy of Science, Warsaw, Poland). The synthesis and identification of the compound were performed as previously described [18].

### 2.2. Cell Culture

Human glioblastoma cell line, U-87 MG, was acquired from Sigma-Aldrich Inc. (St. Luis, MO, USA). Cells were cultured in high-glucose DMEM (Dulbecco’s Modified Eagle Medium) supplemented with 10% heat-inactivated foetal bovine serum, penicillin G (100 U/mL), neomycin (10 μg/mL) and amphotericin B (0.25 µg/mL). Cells were cultured at 37 °C in a 5% CO_2_ humidified environment incubator CB 160 (BINDER GmbH, Tuttlingen, Germany).

### 2.3. Viability and Cell Count Assay

Total cell number and cell viability (percent of viable cells) were assessed using the image cytometer NucleoCunter NC-3000 controlled by the NucleoView NC-3000 Software (ChemoMetec, Lillerød, Denmark). In brief, cells were detached with trypsin/EDTA and the samples of obtained cell suspensions were loaded into the Via1-Cassette (ChemoMetec) containing acridine orange and DAPI.

### 2.4. The Induction of Vitamin B12 Deficiency

U-87 MG cells were seeded into T-25 flasks at a density of 50,000 cells per flask. The cells were cultured in growth medium (4 mL/flask) with HCCL (20 or 50 µg/mL), B12 (50 µg/mL), or HCCL (50 µg/mL) in combination with B12 (50 µg/mL). Every third day, it was replaced with freshly prepared HCCL and/or B12 solution in medium. On day 9 cells were passaged to new T-25 flasks (50,000 cells/flask) and culturing was continued for the next 9 days. Medium samples were collected and centrifuged at 2000× *g* for 20 min. The supernatants were aliquoted and stored at −20 °C until further analysis.

### 2.5. Homocysteine Quantitative Analysis

Homocysteine levels in media samples from control and treated cultures were estimated by quantitative sandwich enzyme-linked immunosorbent assay (ELISA) using Human Hcy ELISA Kit (Abbexa, Cambridge, UK), according to the previously described method [18].

### 2.6. Determination of Cell Cycle Distribution

Cell cycle phase distribution was analyzed by the use of fluorescence image cytometer NucleoCounter NC-3000. The analysis is based on differences in DNA content between the pre-replicative phase (G1/G0) cells versus the cells that actually replicate DNA (S phase) versus the post-replicative plus mitotic (G2/M) cells [25]. In brief, cells were trypsinized, counted, and fixed with ice-cold 70% ethanol. After washing, cell pellets were stained with Solution 3 (ChemoMetec, Lillerød, Denmark) containing DAPI and Triton X-100, loaded into NC-Slides (ChemoMetec), and analyzed using the NC-3000 system where cellular fluorescence was quantified into DNA content histograms. Markers in the displayed histograms were used to identify cells in different cell cycle stages or to demarcate apoptotic cells with fragmented DNA having less than 1 DNA equivalent (sub-G1 cells).

### 2.7. In Vivo Toxicity

The embryos exhibiting normal development were gathered at 0 h post-fertilization (hpf) and transferred into 24-well plates in standard E3 medium (as a control) and a series of concentrations of HCCL solution (10, 50, and 100 μg/mL), until 96 hpf. The experiment was carried out in triplicate and eight embryos were used for each group. The zebrafish embryos were maintained in an environmentally controlled room (26.0 ± 1.0 °C with a light/dark cycle). The embryos were exposed to the test compounds as previously described [26,27] with modifications. Several lethal, sublethal, and teratogenicity points were observed including hatching rate, edema, tail detachment, somite formation, and scoliosis by observation under a stereomicroscope equipped with a camera. The survival rates and morphological deformities of the fertilized eggs were examined and documented at 4, 8, 24, 48, 72, and 96 hpf. The mortality and morphological deformations rates were calculated with GraphPad Prism software.

### 2.8. Molecular Docking

#### 2.8.1. Macromolecule Preparation

The three-dimensional (3D) model of human TCII, also containing two chains of CD320 protein, published by Alam et al. with PDB ID: 4ZRP [28] was retrieved from Protein Data Bank (https://www.rcsb.org/). For the docking study, one polypeptide chain from the crystal structure of TCII homodimer was removed as well as CD320 protein chains. Ligands and water molecules were also eliminated, and hydrogens were added to the structure of TCII. The preparation of the receptor was done in Discovery Studio Visualizer v17.2.0 (Dassault Systems BIOVIA and Discovery Studio Modeling Environment; Release, 2017).

#### 2.8.2. Ligand Preparation

3D structures of HCCL, hydroxocobalamin, methylcobalamin, and adenosylcobalamin were prepared in the Avogadro program [29] basing on the ligand extracted from the cocrystal structure of TCII (4ZRP). To prepare cobalamins before docking, hydrogens were added, and the energy optimization of the models was performed using force field UFF with conjugate gradients algorithm.

#### 2.8.3. Docking of the Ligands

Prepared ligand files were converted in Open Babel [30], part of PyRx v0.8 virtual screening software [31] and loaded into AutoDock Vina [32], which was used for the docking study. The ligand docking was conducted within a grid box covering the cobalamin binding site located in the TCII 3D model with exhaustiveness set at 20. The results of the molecular docking study were visualized in Discovery Studio Visualizer v17.2.0.

### 2.9. Statistics

Statistical significance of differences was tested by one-way ANOVA with Dunnet’s post hoc test based on the results of three independent experiments. Data were graphed and analyzed using GraphPad Prism 8 (GraphPad Software, San Diego, CA, USA). Statistical details can be found directly in the figure captions.

## 3. Results

### 3.1. HCCL Inhibits Glioblastoma Cells Growth

**Figure 1:**
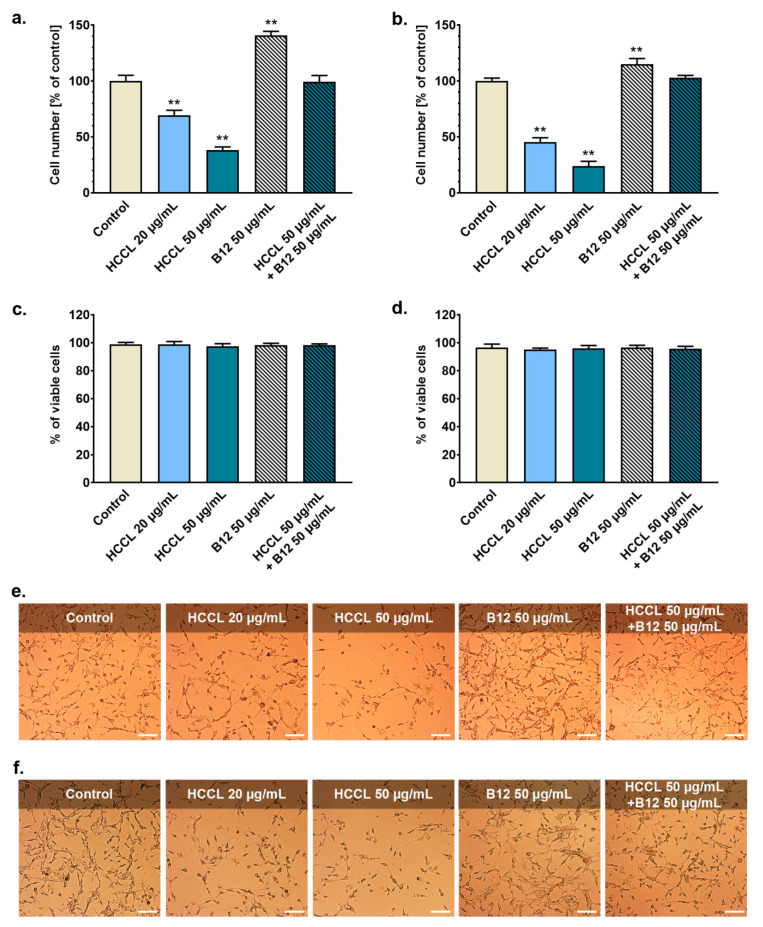
Effect of long-term treatment with B12 antagonist on glioblastoma cells proliferation and viability. U-87 MG cells were cultured with hydroxycobalamin [*c*-lactam] (HCCL) at concentrations of 20 and 50 µg/mL or with vitamin B12 (B12) at a concentration of 50 µg/mL or with HCCL (50 µg/mL) in combination with B12 (50 µg/mL). Untreated cells were cultured in parallel as a control. Results from cell counting on day 9 (**a**) and day 18 (**b**) of the treatment were presented as % of control. Simultaneously, cell viability was assessed after 9 days (**c**) and 18 days (**d**) of culturing. Bars represent mean and SD of three independent experiments in at least triplicate; ** *p* < 0.005 vs. control. Representative microscope images of cells exposed to indicated conditions for 9 days (**e**) and 18 days (**f**); images obtained using light inverted microscope; scale bar = 250 µm.

The culture of U-87 MG glioblastoma cells was examined after exposure to HCCL (20 or 50 µg/mL), B12 (50 µg/mL) or HCCL (50 µg/mL) in combination with B12 (50 µg/mL) for 9 or 18 days. The analysis involved cell counting, viability assay and microscopic imaging. As demonstrated in Figure 1, HCCL and B12 significantly influenced cell proliferation but not viability. After nine days of the experiment, the number of cells in the culture exposed to 20 µg/mL or 50 µg/mL HCCL was reduced by approximately 30% or 62%, respectively (Figure 1a). The antiproliferative effect of HCCL increased with the treatment duration. After 18 days of culturing with 20 µg/mL or 50 µg/mL HCCL, the cell population was reduced by approximately 55% or 76%, respectively, as compared to the control (Figure 1b). In contrast, the cell number in the B12-treated culture exceeded the control by approximately 40% or 14% after 9 or 18 days, respectively. At each time point, the proliferation rate of cells co-treated with HCCL and B12 was at the control level (Figure 1a,b). As shown in Figure 1c,d the percentages of viable cells were >95% under all tested conditions. Analysis of microscopic images obtained on day 9 (Figure 1e) and 18 (Figure 1f) supported the cytometric cell counting data, as a noticeable reduction in the cell number in the HCCL-treated cultures, as well as an increase in confluence in B12-treated cells was observed, revealed a significant reduction in the cell number in HCCL-treated cultures as well as a significant increase in confluence in B12-treated cells. No marked morphological differences were observed between the control and cells treated with HCCL and/or B12.

### 3.2. Treatment with HCCL Enhances Homocysteine Level in Glioblastoma Cell Culture

**Figure 2:**
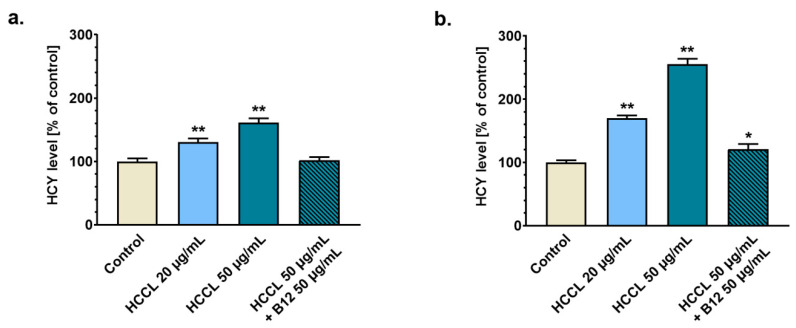
Homocysteine (HCY) level in culture medium after treatment with HCCL and/or B12. U-87 MG cells were cultured with hydroxycobalamin [*c*-lactam] (HCCL) at concentrations of 20 and 50 µg/mL or with HCCL (50 µg/mL) in combination with B12 (50 µg/mL). Untreated cells were cultured in parallel as a control. Data from quantitative analysis of HCY in culture media samples obtained on day 9 (**a**) and day 18 (**b**) of the treatment were calculated for total cell number and presented as % of control. Bars represent mean and SD of three independent experiments in at least triplicate; * *p* <0.05, ** *p* < 0.005 vs. control.

An increase in HCY level reflects a dysfunction in cellular metabolism due to Cbl deficiency [23]. We estimated the HCY level in media from cell culture exposed to HCCL at concentrations of 20 µg/mL and 50 µg/mL. To investigate a putative opposite action of HCCL and B12, we also analyzed media samples from cell culture co-treated with HCCL (50 µg/mL) and B12 (50 µg/mL). As presented in Figure 2a, cell cultures treated with 20 µg/mL or 50 µg/mL HCCL for 9 days had elevated extracellular HCY levels to ca. 130% or 160% of the control value, respectively. We found that this effect increased considerably with a duration of exposure to HCCL. On day 18 of the experiment, the HCY level in the medium of cells treated with 20 µg/mL or 50 µg/mL HCCL was about 1.7- or 2.5-fold higher, respectively, than the control value (Figure 2b). As shown in Figure 2a,b, combined treatment of U-87 MG cells with HCCL and B12 resulted in a complete (in the case of nine-day culture) or partial (in the case of 18-day culture) antagonization of the HCCL-induced HCY accumulation.

### 3.3. HCCL Disturbs Cell Cycle Progression of Glioblastoma Cells

**Figure 3:**
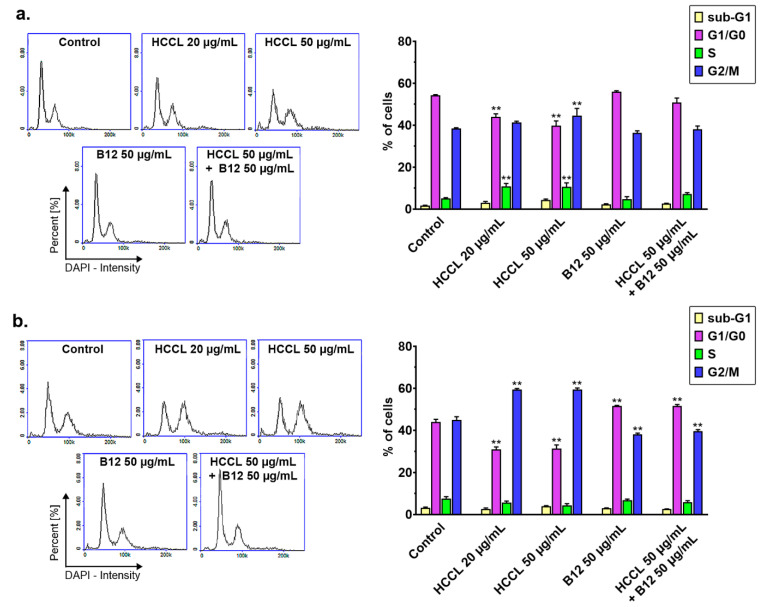
Cell cycle distribution of human glioblastoma cells after treatment with HCCL and/or B12. U-87 MG cells were cultured with hydroxycobalamin [*c*-lactam] (HCCL) at concentrations of 20 and 50 µg/mL or with vitamin B12 (B12) at a concentration of 50 µg/mL or with HCCL (50 µg/mL) in combination with B12 (50 µg/mL). Untreated cells were cultured in parallel as a control. Representative raw histograms (left panel) and statistics (right panel) from cell cycle analysis after 9 days (**a**) or 18 days (**b**) of the treatment. Bars represent mean and SD of three independent experiments in at least triplicate; ** *p* < 0.01 vs. control.

Figure 3 shows the results from the cell cycle assay of U-87 MG cells following 9- and 18-day treatments with HCCL (20 or 50 µg/mL), B12 (50 µg/mL) or HCCL in combination with B12 (each at a concentration of 50 µg/mL). According to Figure 3a, HCCL in both studied concentrations induced a two-fold increase of cell fraction in the S phase when cells were exposed to the agent for 9 days. Moreover, the agent at a concentration of 50 µg/mL caused a slight increase in the percentage of cells in the G2/M phase compared to the control. Combined treatment of U-87 MG cells with HCCL and B12 gave a pattern of cell cycle distribution as for the controls. Similarly, the nine-day cultivation of cells in the only B12-enriched medium did not affect the progression of the cell-division cycle. As shown in Figure 3b, an extension of the treatment time to 18 days resulted in an increase of the percentage of cells in the G2/M phase: from approximately 45% (control level) to approximately 60% (HCCL at both tested concentrations), suggesting the G2/M cell cycle arrest under cobalamin deficiency. There were no significant differences in the fraction of apoptotic cells with fragmented DNA (cells in the sub-G1 phase) between the control and treated cultures.

### 3.4. HCCL Does Not Induce Mortality or Malformations in Zebrafish Embryos

**Figure 4:**
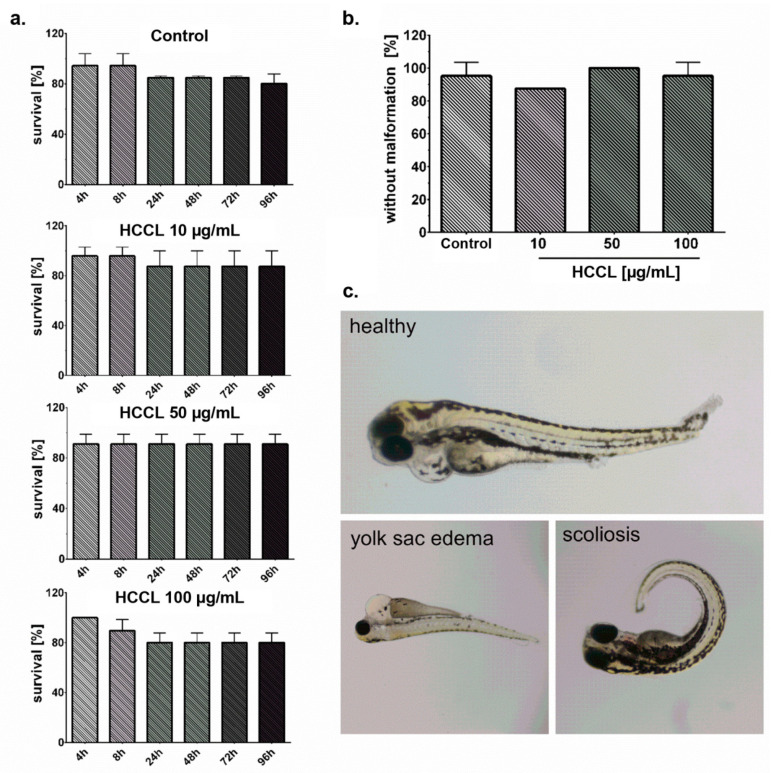
Survival rate of zebrafish exposed to hydroxycobalamin [*c*-lactam] (HCCL) at concentrations of 10, 50, and 100 μg/mL at 4, 8, 24, 48, 72, and 96 hpf (**a**). Malformation rate of zebrafish embryos exposed to HCCL (10, 50, and 100 μg/mL) at 96 hpf (**b**). Representative images of deformed zebrafish exposed to HCCL at 96 hpf (**c**). Bars represent mean and SD, n = 8 from three independent experiments.

Figure 4a shows the survival rates of zebrafish treated with HCCL (10, 50, and 100 μg/mL) at 4, 8, 24, 48, 72, and 96 hpf. The survival rate of zebrafish was concluded based on the presence of heartbeat visual and the absence of a teratogenic effect. There is no significant difference in the incidence of HCCL-induced mortality in zebrafish embryos compared with the control. Additionally, these findings demonstrated low mortality of zebrafish with a survival rate higher than 70%. The typical malformation defects in control embryos and embryos exposed to HCCL were observed in the study, including spinal scoliosis and yolk sac edema (Figure 4c). However, as shown in Figure 4b, there was no significant impact of HCCL on malformation in zebrafish embryos.

### 3.5. HCCL Binds to the Transcobalamin II Active Site as Natural Cobalamins

**Figure 5:**
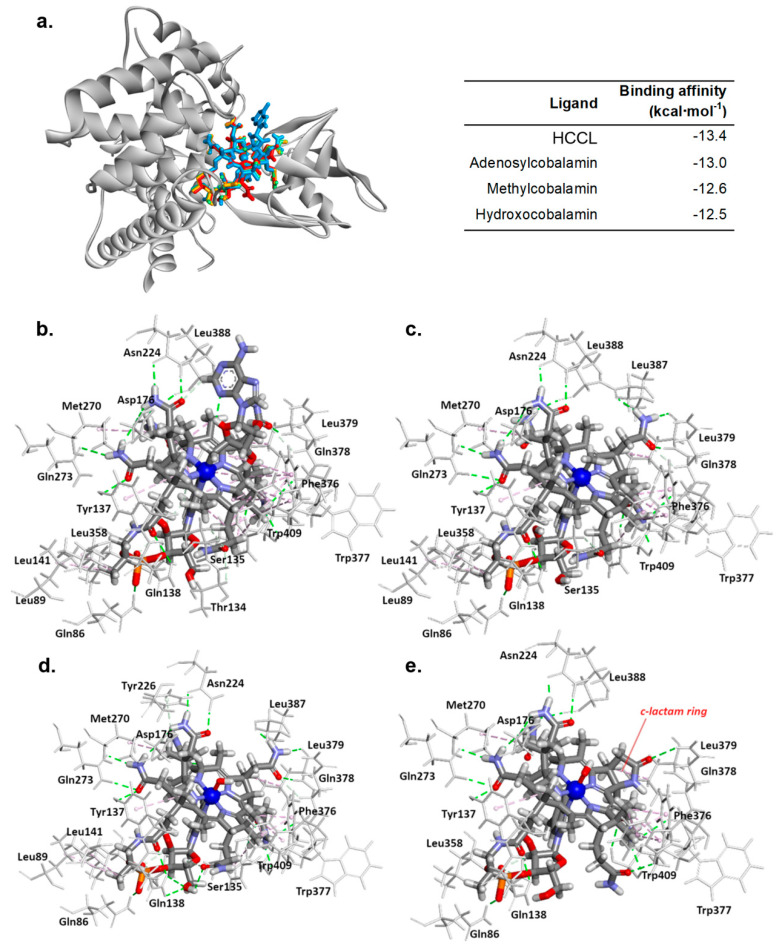
In silico molecular docking results. Docking pose of TCII protein complex with adenosylcobalamin (blue), methylcobalamin (green), hydroxocobalamin (orange), and HCCL (red)—left panel; binding parameters of transcobalamin II-ligand complexes—right panel (**a**). Predicted binding model of adenosylcobalamin (**b**), methylcobalamin (**c**), hydroxocobalamin (**d**), and HCCL (**e**) with transcobalamin II.

We performed in silico analysis to examine the capacity of binding HCCL to TCII. We also carried out the molecular docking of naturally occurring forms of vitamin B12–hydroxocobalamin, methylcobalamin, and adenosylcobalamin [33] for comparison purpose. The docking study showed that HCCL binds to the TCII active site in a similar manner to natural cobalamins (Figure 5). Among the ligands used in the in silico study, the lowest binding energy to TCII was exhibited by HCCL and it was calculated to be −13.4 kcal∙mol^−1^. The docking study also revealed that this compound shares many ligand-TCII interactions with the naturally occurring cobalamins (Table S1). However, there are some interactions that are not present in the HCCL-TCII complex (Table S2), which are formed with Leu89, Leu141, Leu379, and Tyr362 residues of the protein in the complexes with other analyzed cobalamins. There are also some interactions unique for HCCL concerning different chemical groups than in other compounds from the study, i.e., γ-lactam ring forming a hydrogen bond with Gln378 residue and propamide group forming a hydrogen bond with Trp409 amino acid.

## 4. Discussion

The clinical results are often disappointing and conventional chemotherapies continue to fail in treating many types of cancers, including glioblastoma. New effective therapeutic strategies must be developed in this field, and the metabolic abnormalities of cancer cells offer such opportunities. According to Zelder et al. [6], Cbl antagonists offer the enormous medicinal potential for the development of anticancer agents and further development is urgently required to push antivitamins B12 research to the next level, from academic research into clinical trials. The potential benefits of the induction of Cbl deficiency in cancer cells result from the fact that the key function of B12 is to accomplish DNA synthesis, which is essential for cell proliferation. Moreover, cancer cells exert much higher demand for B12 than normal cells, which makes Cbl transport and metabolism an interesting target for anticancer therapy [5,6]. On the other hand, the state of vitamin deficiency may lead to cancerogenesis via induction of mutagenic DNA lesions such as point mutation, single and double-stranded DNA breaks, chromosome breakage, as well as alteration of DNA methylation [22]. Thus, the effect of B12 status on cancer progression still needs to be investigated.

Here, for the first time, we determine the impact of B12 status on glioblastoma at the cellular level. In the first step, we developed a stable in vitro model of Cbl deficiency in U-87 MG cells. To inhibit biochemical pathways related to B12 in glioblastoma cells, we applied long-term treatment of the cells with cobalamin antagonist-HCCL. HCCL has previously been found as an effective Cbl antagonist in several in vitro [14,15,16] and in vivo studies [12,13,17]. For example, in the study by Sauer et al. [15], treatment of human proximal tubule cells with HCCL resulted in an inhibition of cobalamin-dependent metabolism and an increase of the level of hypocobalaminemia biomarkers, i.e., homocysteine and methylmalonic acid. Similar effects were obtained on rat oligodendrocytes [14] and hepatocytes [16]. In addition, Stabler et al. [13] and Haegler et al. [17] revealed, that administration of HCCL in rodents led to severe metabolic disorders analogous to cobalamin deficiency.

When developing an experimental model of Cbl deficiency based on HCCL treatment, it is necessary to adjust the culture conditions for individual types of cells. This is due to the various demand of cells for B12, as well as the diverse content of Cbl in the culture media. Previously, we developed an in vitro model of hypocobalaminemia in normal human melanocytes [18] and normal human astrocytes [19] by exposing the cells to HCCL at a concentration of 10 µg/mL for 24 days or 20 µg/mL for 27 days, respectively. Here, the proposed experimental model assumes the treatment of U-87 MG glioblastoma cells with HCCL in concentrations of 20 and 50 µg/mL for 18 days. The treatment resulted in a significant increase in extracellular HCY levels, indicating that the state of Cbl deficiency was obtained. Moreover, the addition of vitamin B12 to the culture medium prevented the excess of HCY produced by the cells, suggesting the antagonistic activities of HCCL and the vitamin.

Our data indicated a significant time- and dose-dependent cytostatic effect of HCCL on glioblastoma cells. Interestingly, the viability of treated cells was not affected under any of the tested conditions. The 18-days culture of U-87 MG cells with HCCL at a concentration of 20 µg/mL resulted in a significant reduction of cell proliferation by approximately 55% when compared to the control. In the case of normal astrocytes treated with HCCL in a concentration of 20 µg/mL for 27 days, the inhibition of cell proliferation by 20% was observed [19]. We hypothesized that the need for a longer exposure to the antagonist to produce a similar deficiency effect in normal astrocytes may be due to the fact that astrocytes have a lower consumption rate of Cbl than their cancer counterparts. The obtained results may also be confronted with the study presented by Sponne et al. [14], where no significant anti-proliferative effect was observed in normal rat oligodendrocytes exposed to HCCL (5 and 10 µg/mL) for 30 days.

In the present study, when U-87 MG cells were simultaneously treated with HCCL and B12, cell proliferation was at the control level, suggesting opposed activities of these two agents. Moreover, we demonstrated that B12 strongly promoted glioblastoma cells proliferation. Interestingly, as the duration of the experiment increased, the stimulating effect of Cbl diminished. A similar observation was described by Evans et al. [34] on L-929 cells. They indicated that B12 at a concentration of 50 μg/mL increased cell proliferation in the first week of the culture, but the elevated rate of proliferation was not maintained for the remaining time of the experiment. Since intracellular Cbl uptake is strictly dependent on TCII and TCblR [5], we suggest, that the lack of intensification of the proliferation stimulating effect with increasing duration of the B12 treatment may be due to TCblR oversaturation and insufficient level of TCII. Indeed, it has been demonstrated, that TCblR densities on malignant cells (K562 and HL-60) correlated inversely with the concentration of Cbl in the culture medium, suggesting that intracellular stores of Cbl may affect the expression of TCblR [35].

Several studies on experimental animals or cultured cells have shown that treatment with HCCL may lead to the blockage of B12-dependent metabolic processes [12,13,14,15,16,17,18,19]. However, it is not known at what stage the blockage occurs since the cellular and molecular mechanism of HCCL action has not yet been fully elucidated. Stabler et al. [13] in the in vivo study on rats revealed that HCCL treatment resulted in a significant decrease of mean liver holo-L-methylmalonyl-coenzyme A mutase and MS activity and greatly increased serum methylmalonic acid and HCY concentrations. According to Zelder et al. [6], HCCL belongs to the class A2 of antivitamins B12 and most probably, the inhibitory effect of the agent is due to blocking of the intracellular uptake and/or subsequent metabolic conversion of cobalamins rather than directly inhibiting the vitamin-dependent enzymes. This statement is in good agreement with our data from the molecular docking study presented here. We have demonstrated that HCCL shares similar interactions with TCII as naturally occurring cobalamins and thus it might act as a competitive inhibitor of this transporter protein. Further, it is probable that the cellular uptake of the HCCL-TCII complex is not affected by this compound, because the in silico study did not show any interactions between the amino acids of the CD320 receptor binding domain of TCII (i.e., His56, His104, Lys105, Lys114, Trp115, Glu118, Arg122, and His154 [28]). However, further studies are needed to confirm this hypothesis.

Cell division is controlled by multiple checkpoint mechanisms that block transitions between cell-cycle phases when cells encounter stressful conditions [36]. Cells with alarmed checkpoints can be eliminated by apoptosis or silenced by cellular senescence or can survive and resume cell cycle progression after the defects have been repaired [37]. Our data from the viability and cell count cytometric assay supported by the microscopic observations prompted us to conduct a cell cycle analysis to explore the strong cytostatic effect of HCCL on U-87 MG cells. Our data indicated that the Cbl antagonist significantly affected cell cycle progression and vitamin B12 counteracted the HCCL effects, as in the case of cell proliferation and HCY accumulation. There was no significant increase of cells in the sub-G1 phase under all conditions tested, indicating that the treatments did not induce apoptosis. These results are in agreement with data from the viability assay. After 9 days of the treatment with the agent, we observed a slight increase of cells in the S phase. This may have been the result of the activation of S-phase checkpoints. The cell cycle can be arrested here if the DNA is unreplicated, incomplete, or damaged, and therefore the cell is not competent to proceed to phase G2 [38,39]. Due to the fact that vitamin B12 and folate are very closely connected in the metabolism, our results are worth comparing with the results obtained by Chen et al. [40]. They revealed that human lung adenocarcinoma A549 cells treated with pemetrexed, an antifolate drug approved for lung cancer therapy, showed accumulation in the S phase of the cell cycle. The S-phase arrest was also observed in human prostate carcinoma cells PC-3 cultured in methionine-free, HCY-containing medium [41]. Regeneration of methionine from HCY is catalyzed by MS, and the reaction links both Cbl and folate metabolism. The disturbance of cell homeostasis associated with methionine restriction and Cbl deficiency may be similar. It has been shown that cancer cells require elevated amounts of methionine and arrest their growth under conditions of methionine restriction. As shown by Hoffman et al. [41], in addition to S-phase arrest, the methionine deficiency also led to the majority of the cancer cells being blocked in the G2 phase of the cell cycle [41]. Similarly, in our study, we found that cells were arrested in the G2/M phase when treated with HCCL for 18 days. The arrest may explain the significant decrease in cell proliferation observed since the G2/M checkpoint is a key cell cycle checkpoint that prevents cells from entering mitosis until damaged or incompletely replicated DNA is sufficiently repaired [36].

The zebrafish is now firmly established as a powerful research in vivo model for assessing human risk and for preclinical drug discovery and screening [42]. In light of our in vitro results, we determined the effect of HCCL on zebrafish embryos. It should be noted that even at a concentration higher than the concentration significantly disturbing the glioblastoma cells culture, HCCL had no significant impact on mortality or developmental malformations of zebrafish embryos. These findings may promote further research using HCCL.

## 5. Conclusions

Taken together, our findings have extended understanding of the impact of vitamin B12 status on glioblastoma at the cellular level. We revealed that the induction of vitamin B12 deficiency by HCCL inhibited the growth of glioblastoma cells, mainly by G2/M cell cycle arrest. The effect was observed for the agent concentration non-toxic for the zebrafish. Moreover, as compared to the previous study, the cytostatic effect of HCCL was more pronounced in glioblastoma cells than in normal astrocytes. We believe that our findings may become the basis for further in vitro and in vivo experiments concerning cobalamin deprivation as a potential therapeutic strategy for glioblastoma.

## Supplementary Materials

The following are available online at https://www.mdpi.com/2079-7737/10/1/69/s1, Table S1: Interactions between transcobalamin II (TCII) and hydroxycobalamin *c*-lactam (HCCL) or natural cobalamins common for every ligand used in the molecular docking study, Table S2: Differences in the interactions between transcobalamin II (TCII) and ligands used in the molecular docking study.
