# Supplementary material for: Response of Human Glioblastoma Cells to Vitamin B12 Deficiency: A Study Using the Non-Toxic Cobalamin Antagonist
Source: Biology (Basel). 2021 Jan 19;10(1):69. doi: 10.3390/biology10010069 (PMC7835758; doi:10.3390/biology10010069)
Supplement: Supplementary file 1 [file biology-10-00069-s001.pdf]

Table S1: Interactions between transcobalamin II (TCII) and hydroxycobalamin [*c*-lactam] (HCCL) or natural cobalamins common for every ligand used in the molecular docking study.

TCII	HCCL			Hydroxycobalamin			Methylcobalamin			Adenosylcobalamin		
**Residue**	**Residue**	**Type**	**Length [Å]**	**Residue**	**Type**	**Length [Å]**	**Residue**	**Type**	**Length [Å]**	**Residue**	**Type**	**Length [Å]**
**Asn224**	acetamide	conventional hydrogen bond	1.96	acetamide	conventional hydrogen bond	2.06	acetamide	conventional hydrogen bond	2.02	acetamide	conventional hydrogen bond	2.21
	acetamide	conventional hydrogen bond	2.20	acetamide	conventional hydrogen bond	2.11	acetamide	conventional hydrogen bond	2.16	acetamide	conventional hydrogen bond	2.23
**Asp176**	acetamide	conventional hydrogen bond	2.02	acetamide	conventional hydrogen bond	2.00	acetamide	conventional hydrogen bond	2.06	acetamide	conventional hydrogen bond	2.03
	acetamide	conventional hydrogen bond	2.62	acetamide	conventional hydrogen bond	2.43	acetamide	conventional hydrogen bond	2.48	acetamide	conventional hydrogen bond	2.60
**Gln138**	carbonyl	conventional hydrogen bond	2.44	carbonyl	conventional hydrogen bond	2.6	carbonyl	conventional hydrogen bond	2.74	carbonyl	conventional hydrogen bond	2.95
**Gln273**	acetamide	conventional hydrogen bond	2.18	acetamide	conventional hydrogen bond	2.44	acetamide	conventional hydrogen bond	2.33	acetamide	conventional hydrogen bond	2.71
**Gln86**	phosphate	conventional hydrogen bond	1.64	phosphate	conventional hydrogen bond	1.83	phosphate	conventional hydrogen bond	1.70	phosphate	conventional hydrogen bond	1.56
**Leu358**	phosphate	conventional hydrogen bond	2.07	phosphate	conventional hydrogen bond	2.09	phosphate	conventional hydrogen bond	2.00	phosphate	conventional hydrogen bond	1.92
**Leu363**	propamide	conventional hydrogen bond	2.29	propamide	conventional hydrogen bond	2.11	acetamide	conventional hydrogen bond	1.90	propamide	conventional hydrogen bond	2.12
	propamide	conventional hydrogen bond	2.71	propamide	conventional hydrogen bond	2.68	propamide	conventional hydrogen bond	2.26	propamide	conventional hydrogen bond	2.65
**Leu379**	methyl	alkyl	4.71	methyl	alkyl	4.60	methyl	Alkyl	4.48	methyl	alkyl	4.52
**Met270**	methyl	alkyl	4.66	methyl	alkyl	4.75	methyl	Alkyl	4.66	methyl	alkyl	5.02
**Phe376**	methyl	π-alkyl	3.41	methyl	π-alkyl	3.70	methyl	π-alkyl	3.84	methyl	π-alkyl	4.00
	methyl	π-alkyl	3.94	methyl	π-alkyl	3.84	methyl	π-alkyl	3.85	methyl	π-alkyl	4.17
**Ser357**	phosphate	carbon hydrogen bond	2.52	phosphate	carbon hydrogen bond	2.65	phosphate	carbon hydrogen bond	2.73	phosphate	carbon hydrogen bond	2.74
**Trp409**	methyl	π-alkyl	2.62	methyl	π-alkyl	4.45	methyl	π-alkyl	4.24	methyl	π-alkyl	3.80
	methyl	π-alkyl	4.85	methyl	π-alkyl	4.50	methyl	π-alkyl	4.94	methyl	π-alkyl	4.88
**Tyr137**	acetamide	conventional hydrogen bond	2.41	acetamide	conventional hydrogen bond	2.42	acetamide	conventional hydrogen bond	2.54	acetamide	conventional hydrogen bond	2.69
	methyl	π-alkyl	4.65	methyl	π-alkyl	4.71	methyl	π-alkyl	4.78	methyl	π-alkyl	4.87

Table S2: Differences in the interactions between transcobalamin II (TCII) and ligands used in the molecular docking study.

TCII	HCCL			Hydroxycobalamin			Methylcobalamin			Adenosylcobalamin		
Residue	Residue	Type	Length [Å]	Residue	Type	Length [Å]	Residue	Type	Length [Å]	Residue	Type	Length [Å]
Gln138	-	-	-	hydroxyl	conventional hydrogen bond	2.74	-	-	-	-	-	-
	-	-	-	hydroxyl	conventional hydrogen bond	2.49	-	-	-	-	-	-
Gln273	acetamide	conventional hydrogen bond	2.41	acetamide	conventional hydrogen bond	2.19	acetamide	conventional hydrogen bond	2.70	-	-	-
Gln378	γ-lactam ring	conventional hydrogen bond	2.70	-	-	-	-	-	-	-	-	-
	-	-	-	acetamide	carbon hydrogen bond	2.67	acetamide	carbon hydrogen bond	2.47	acetamide	carbon hydrogen bond	2.95
Gly390	-	-	-	propamide	conventional hydrogen bond	2.54	-	-	-	propamide	conventional hydrogen bond	2.57
Leu141	-	-	-	methyl	alkyl	5.39	methyl	alkyl	5.27	methyl	alkyl	5.25
Leu358	-	-	-	-	-	-	-	-	-	methyl	alkyl	5.41
Leu379	-	-	-	acetamide	conventional hydrogen bond	2.18	acetamide	conventional hydrogen bond	2.08	acetamide	conventional hydrogen bond	1.85
	-	-	-	acetamide	conventional hydrogen bond	2.18	acetamide	conventional hydrogen bond	1.88	-	-	-
Leu387	-	-	-	acetamide	conventional hydrogen bond	1.91	acetamide	conventional hydrogen bond	2.07	-	-	-
Leu388	propamide	conventional hydrogen bond	2.26	-	-	-	propamide	conventional hydrogen bond	2.22	propamide	conventional hydrogen bond	2.91
Leu89	-	-	-	methyl	alkyl	5.25	methyl	alkyl	4.92	methyl	alkyl	4.62
Phe376	-	-	-	-	-	-	-	-	-	pyrolidine	π-alkyl	5.07
	-	-	-	-	-	-	-	-	-	pyroline	π-alkyl	5.40
	-	-	-	-	-	-	-	-	-	propamide	π-alkyl	5.10
Ser135	-	-	-	-	-	-	propamide	carbon hydrogen bond	2.56	propamide	carbon hydrogen bond	2.60
Thr134	-	-	-	-	-	-	-	-	-	propamide	carbon hydrogen bond	2.98

**Trp377**	propamide	conventional hydrogen bond	2.60	propamide	conventional hydrogen bond	2.71	-	-	-	propamide	conventional hydrogen bond	2.77
	-	-	-	-	-	-	acetamide	conventional hydrogen Bond	2.50	-	-	-
**Trp409**	propamide	conventional hydrogen bond	2.49	-	-	-	-	-	-	-	-	-
	methyl	π-alkyl	1.96	-	-	-	methyl	π-alkyl	4.23	methyl	π-alkyl	3.80
	-	-	-	-	-	-	-	-	-	pyroline	π-alkyl	5.23
**Tyr226**	-	-	-	propamide	π -donor hydrogen bond	2.95	-	-	-	-	-	-
**Tyr362**	-	-	-	methyl	π-alkyl	5.33	methyl	π-alkyl	5.30	methyl	π-alkyl	5.23
	-	-	-	-	-	-	-	-	-	methyl	π-alkyl	5.10
